# Lipoid Proteinosis

**DOI:** 10.5005/jp-journals-10005-1353

**Published:** 2016-06-15

**Authors:** Hitesh C Mittal, Sunil Yadav, Sunita Malik, Gurdarshan Singh

**Affiliations:** 1 Senior Resident, Department of Dentistry, BPS Government Medical College for Women, Khanpur Kalan, Haryana, India; 2 Professor and Head, Department of Dentistry, BPS Government Medical College for Women, Khanpur Kalan, Haryana, India; 3 Assistant Professor, Department of Dentistry, BPS Government Medical College for Women, Khanpur Kalan, Haryana, India; 4 Demonstrator, Department of Dentistry, BPS Government Medical College for Women, Khanpur Kalan, Haryana, India

**Keywords:** Lipoid proteinosis, Recurrent vesicular oral ulcers, Urbach-Wiethe disease.

## Abstract

A case report of a 6-year-old male child who reported with recurrent oral and skin ulcerations since childhood and was diagnosed as lipoid proteinosis manifesting with generalized thickening, hardening, and scarring of the skin and hoarseness of voice; is presented here.

**How to cite this article:** Mittal HC, Yadav S, Malik S, Singh G. Lipoid Proteinosis. Int J Clin Pediatr Dent 2016;9(2):149-151.

## INTRODUCTION

Lipoid proteinosis, a very rare autosomal recessive geno-dermatosis, results in hyaline material deposition in the skin and mucous membrane of various organs leading to multisystem involvement.^1^ Lipoid proteinosis is also known as Urbach-Wiethe disease or Hyalinosis cutis et mucosae. Lipoid proteinosis is caused by loss of function mutations in the extracellular matrix protein 1 gene (ECM1) on 1q21.^2^ The exact function of ECM1 gene is not known. But it is supposed to have important physiological and biological actions in epidermal differentiation, binding of dermal collagens and proteoglycans, and regulation of angiogenesis. Mutations in the ECM1 gene result in abnormalities in the degradation pathway of glycolipids or sphingolipids, underproduction of the fibrous collagens, and overproduction of basal membrane collagens.^2,3^ With about only 300 cases reported till now,^1^ including both from live patients and cadaver specimens, the details of etiopathogenesis, course of disease, treatment modalities with long-term follow-up, and prognosis are still debatable.

The purpose of this article is to report on a 6-year-old male child manifesting classical features of lipoid proteinosis who actually reported for recurrent oral ulcerations since childhood.

## CASE REPORT

A 6-year-old male patient reported to the Department of Dentistry with complaints of frequent ulcers in the mouth. On history, patient reported hoarseness of voice and recurrent spontaneous erosions all over the body which healed by hypo- to depigmented scars since 6 months of age. History of pruritus on and off was also present. History of recurrent oral erosions was present since 2 years of age. Patient took various treatments in the form of steroids and antibiotics without any improvement. No history of seizures, cognitive or behavioral impairment, hypertrophic scarring, visual impairment, stridor, respiratory difficulties, and dysphasia was present. Family history revealed that the child was born of nonconsanguineous marriage. No prenatal and natal complications were present. None of her family members or relatives had a similar condition. Patient had normal developmental milestones. The two other siblings were normal.

On general examination, patient was cooperative, well oriented to time, place, and person. However, he was of average build and had low and squeaky voice. No pallor, icterus, cyanosis, pedal edema, lymphadenopathy, organo-megaly were present. No other systemic abnormality was detected.

**Fig. 1:**
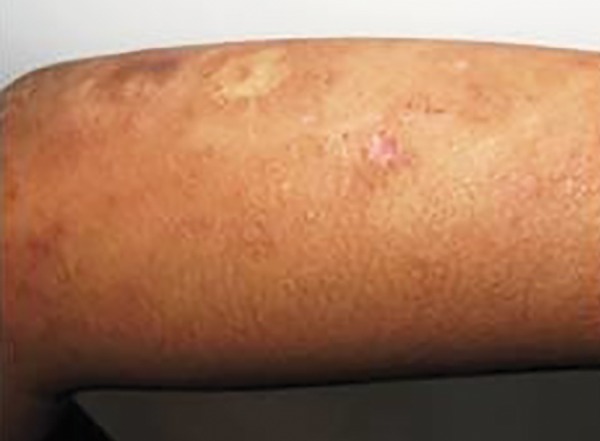
Hyper/hypo/depigmented scars on left arm

**Fig. 2:**
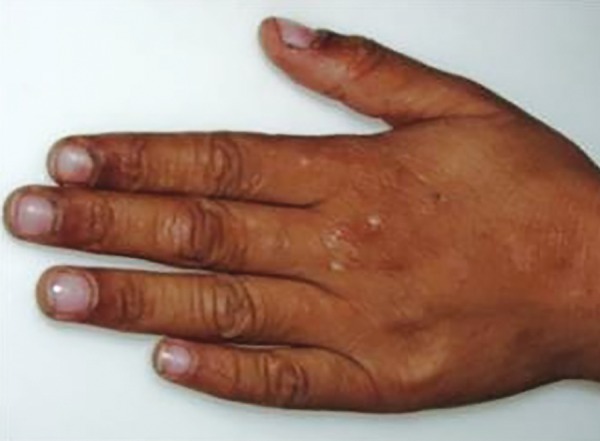
Hyper/hypo/depigmented scars on left hand

**Fig. 3:**
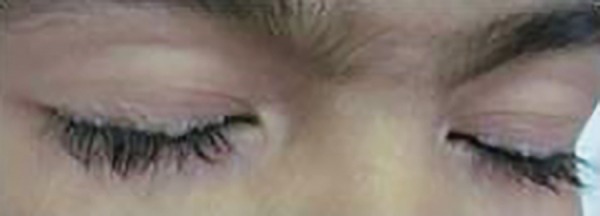
Moniliform blepharosis on eyelids

On extraoral examination, few well-defined crusted types of erosion over dorsum of hands and elbows and hyper-, hypo-, or depigmented scars were also present over back, arms, elbows, and dorsum of hands (Figs 1 and 2). The characteristic beaded papules, also known as “moniliform blepharosis,” were present on eyelids of both eyes (Fig. 3).

**Fig. 4:**
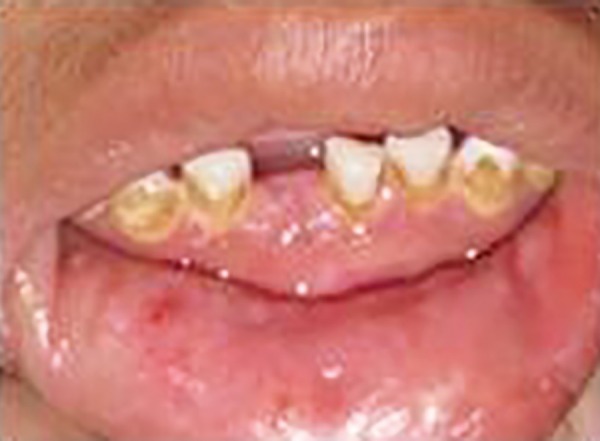
Small isolated vesicular ulcerations and thickening of labial mucosa

**Fig. 5:**
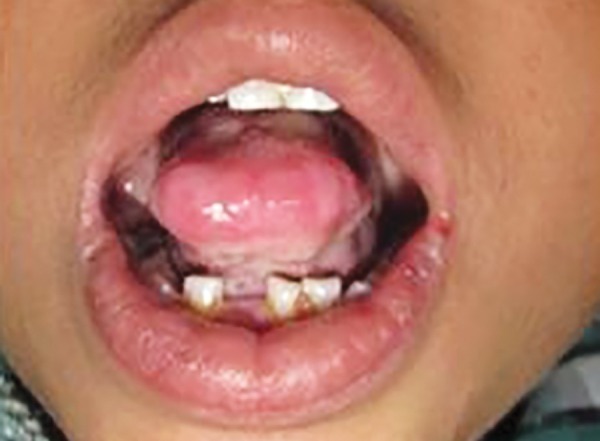
Thickened frenulum with inability to protrude out the stiff tongue completely

**Fig. 6:**
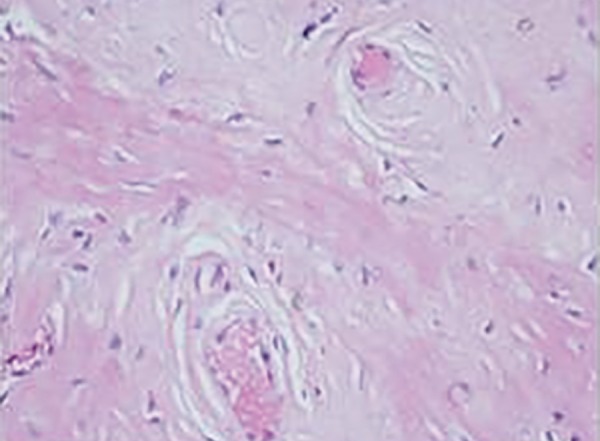
Histopathological examination slide

On intraoral examination, mouth opening was restricted without any abnormalities regarding temporo-mandibular joint, which suggested that normal skin elasticity required for mouth opening was lacking. Soft tissue examination revealed pale mucosa and showed irregular elevations and depressions with pearly nodular deposits throughout. It was hard and indurated. Small isolated vesicular ulcerations were visible on oral mucosa (Fig. 4). Lips were thickened and infiltrated. Tongue was stiff and frenulum thickened with inability to protrude out the tongue completely. The lateral border showed clear demarcations of dentition, indicating macroglossia (Fig. 5).

The clinical findings along with the histopathological examination of oral mucosa (Fig. 6) confirmed the diagnosis of lipoid proteinosis. Treatment was started with a second-generation retinoid, Acitretin 10 mg/day. He showed mild improvement in occurrence of erosions and reduction in scarring. Patient was also referred to ear, nose, and throat and dermatology department where he was put on speech therapy and emollients were prescribed.

## DISCUSSION

Lipoid proteinosis is a very rare, autosomal recessive disorder, characterized by infiltration of hyaline material into the skin, oral cavity, larynx, and internal organs. The most important clinical features are hoarseness of voice and thickened sublingual frenulum, which prevents the patients from protruding their tongue and hence the clinical admonition “listen to them talk and have them stick out their tongues.” Skin lesions usually develop within first few years of life, though the timing of their onset is variable. The first skin lesions are often blisters in early childhood, which become eroded and crusted after minor trauma. The scarring of these erosions usually begins during childhood and is often worst in the face. It may follow trauma or occur spontaneously. Pox-like or acneiform scarring is particularly evident in the face and extremities. The classic and most easily recognizable sign is the beaded eyelid papules (moniliform blepharosis), although the papular infiltration may be quite subtle in some patients.^1-8^

The mucosa of the patients exhibits infiltration and thickening. Oral erosions may be noted early in life. Thickening of lingual frenulum leads to inability to protrude the tongue. The pharynx, tongue, soft palate, lips, and tonsils are typically involved. Infiltration of Stensen’s duct and its blockage may lead to parotitis. Xerostomia and dental abnormalities may also be seen. Other findings include seizures, memory deficits, social, and behavioral changes, paranoid symptoms, mental retardation, and aggressiveness. Generalized dystonia has also been reported. Eyelid infiltration may induce corneal ulcers due to trichiasis. There also may be loss of eyelashes.^1,4^

The histopathological features include epidermal hyperkeratosis, irregular acanthosis, thickened dermis, and presence of large deposits of periodic acid Schiff-positive and diastase-resistant extracellular hyaline material. Ultrastructural findings include multiple concentric rings of basement membrane around blood vessels and irregular reduplication of the lamina densa at the dermal-epidermal junction.^3^

Erythropoietic protoporphyria, epidermolysis bullosa, and impetigo should be considered in the differential diagnosis of lipoid proteinosis. Erythropoietic protoporphyria may display similar skin symptoms, but not oral lesions.^5^ Increased values of protoporphyrin in erythrocytes are a key symptom.

There is no effective treatment available, although oral application of dimethyl sulfoxide,^6^ oral etretinate,^7^ and D-penicillamine^8^ have been tried in literature. The prognosis is variable. Usually lipoid proteinosis has a benign course with a normal life expectancy of patients. There is progressive worsening of cutaneous features with clearance of blistering lesions in childhood and progressive development of infiltrative lesions. The hoarseness and mucosal lesions may progress with time.

## CONCLUSION

The knowledge of the clinical features of the disease may help the oral health professional in rendering the appropriate treatment in order to improve the quality of life deteriorated by the disease.
